# Synchronous Ovarian and Endometrial Endometrioid Adenocarcinoma Presenting with Nonbacterial Thrombotic Endocarditis and Pulmonary Thromboembolism: Adenocarcinoma with Thrombotic Events

**DOI:** 10.1155/2015/825404

**Published:** 2015-10-21

**Authors:** N. K. Erturk, A. Erturk, D. Basaran, N. Ozgul

**Affiliations:** ^1^Department of Gynecology and Obstetrics, Hacettepe University Faculty of Medicine, 6530 Ankara, Turkey; ^2^Division of Gynecologic Oncology, Etlik Zubeyde Hanim Research Hospital, 6530 Ankara, Turkey; ^3^Division of Gynecologic Oncology, Hacettepe University Faculty of Medicine, 6530 Ankara, Turkey

## Abstract

*Introduction.* Nonbacterial thrombotic endocarditis (NBTE) is a rare manifestation of hypercoagulability in patients with malignant neoplasms. *Case Report.* A fifty-six-year-old woman presented to the emergency service; the clinical workup revealed deep vein thrombosis in right leg and bilateral massive PTE. As the abdominal sections on the spiral CT revealed a giant pelvic mass of ovarian origin, she was referred to our hospital's gynecologic oncology department. She was scheduled for surgery under enoxaparin. She described numbness on one side of her face. Cranial imaging findings revealed acute ischemic cerebral lesions and transesophageal echocardiogram showed vegetation on the aortic cusp. Under anticoagulation treatment, she underwent hysterectomy with bilateral salpingo-oophorectomy and infracolic omentectomy. After tumor resection, her neurological symptoms dissolved with aggressive anticoagulant treatment. Pathology result was synchronous endometrial and ovarian adenocarcinoma. *Discussion.* NBTE is a rare condition often associated with advanced malignancies. Peripheral embolism and venous thrombosis are complications that have been associated with NBTE due to hypercoagulable state. These disorders could be resistant to routine anticoagulant treatment. In case of a thrombotic complication due to ovarian malignancy, surgical resection of the primary tumor may increase the effect of anticoagulant treatment.

## 1. Introduction

Thrombosis is a common complication in cancer patients, with 15% of all cancer patients developing clinically apparent thrombosis [1]. Systemic coagulopathy underlies the thrombosis in patients with malignant disease. Hypercoagulability of malignancy could represent a clinical spectrum ranging from abnormal coagulation tests, but no clinically evident thromboembolic disease, to arteriovenous thrombosis, migratory thrombophlebitis, nonbacterial thrombotic endocarditis, and disseminated intravascular coagulation [2].

Nonbacterial thrombotic endocarditis (NBTE) is a rare manifestation of hypercoagulability in patients with malignant neoplasms. Peripheral embolism and venous thromboses are complications that have been associated with NBTE due to a hypercoagulable state [3]. Systemic embolization to multiple organs is common, with embolization to brain ranging from 14 to 91% [4].

Here, we report a rare association of NBTE in a patient with two primary gynecological cancers presenting with embolic stroke and pulmonary thromboembolism (PTE). To the best of our knowledge, such a case has never been reported in the literature.

## 2. Case Report

**Figure 1:**
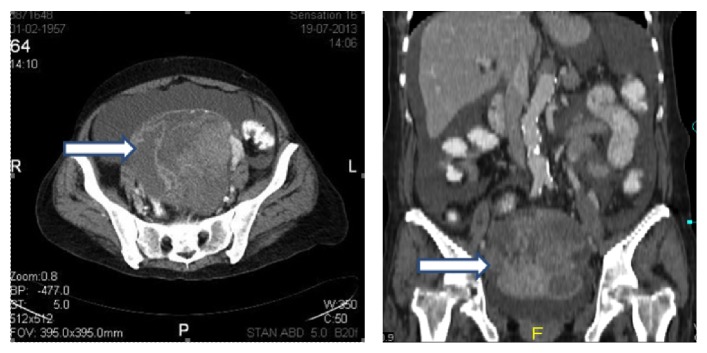
Abdominal computerized tomography revealed a complicated pelvic mass of ovarian origin (arrows).

A fifty-six-year-old nulligravid woman was admitted to the emergency service of a community hospital with shortness of breath and mild chest pain. The clinical workup revealed deep vein thrombosis in her right leg and bilateral massive PTE. As the abdominal sections on the spiral computerized tomography (CT) scan revealed a giant pelvic mass of ovarian origin with septa, solid component, and ascites, she was referred to our hospital's gynecologic oncology department after initial anticoagulant treatment with unfractionated intravenous (IV) heparin. Her initial examination was remarkable for pelvic mass up to the level of umbilicus and mild orthopnea. In the repeated abdominal CT scan, a 165 × 130 × 90 mm solid cystic mass probably originated from the left ovary, ascites, and splenic infarcts were seen (Figure 1). Her CA-125 value was 23000 U/mL, fibrinogen was 795 mg/dL, and D-Dimer was 20 mg/dL. After consultation with our pulmonology department, she was scheduled for surgery under enoxaparin 0.8 mL b.i.d.

**Figure 2:**
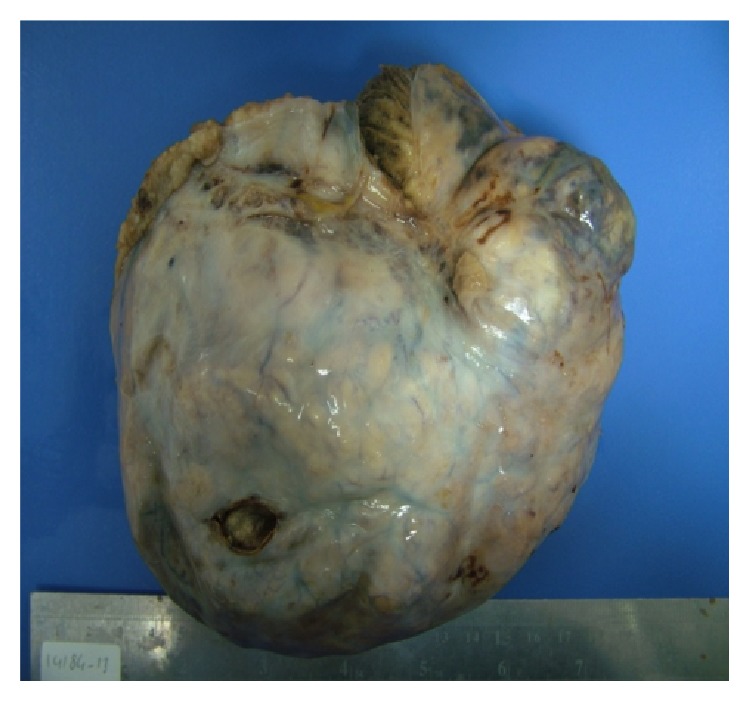
Pelvic mass of ovarian origin after operation.

Two days before the planned day of surgery, the patient described numbness on one side of her face. The patient was examined by a neurologist and a cranial CT scan was found to be normal. In the night before surgery, the patient developed sudden aphasia and dizziness. In the cranial diffusion MRI, there were multiple lesions at the border zones and there was a huge acute infarct at right temporal zone. She was started on IV heparin treatment immediately after the diagnosis. Transesophageal echocardiogram (TEE) showed 7 × 2 mm vegetation on the mitral cusp. Despite IV heparin treatment for almost 48 hours, her neurological examination did not improve and got worsened. The patient became lethargic and she started to have episodic seizures. After an urgent bedside consultation with the neurology department and having informed consent from the patient, she was taken to the operation room and underwent hysterectomy with bilateral salpingo-oophorectomy and infracolic omentectomy under anticoagulation treatment with full dose IV heparin (Figure 2). After tumor resection, her neurological symptoms dissolved gradually with ongoing anticoagulant treatment.

Pathology result was synchronous stage IA, grade 2, endometrioid endometrial adenocarcinoma and stage IC grade 2 endometrioid ovarian adenocarcinoma. She received six cycles of platinum based adjuvant chemotherapy. At one-year follow-up, her abdominal CT scan showed no evidence of disease and her aphasia almost disappeared with speech therapy.

## 3. Discussion

Over 90% of cancer patients develop laboratory evidence of coagulation abnormalities during the course of a malignant illness or its treatment [5]. Increased platelet aggregation, tumor-cell procoagulants, and increased levels of coagulation factors are the mechanisms of paraneoplastic hypercoagulability [6]. Tumor necrosis factor and interleukins that are released from malignant cells can cause endothelial damage, converting the surface of the heart valves to a thrombogenic surface [7].

In case of a malignancy, procoagulant activity increases and fibrinolytic activity decreases. This combination accelerates the prothrombotic potential of endothelial cells [8].

NBTE is a rare condition often associated with hypercoagulable states or advanced malignancies and is characterized by cardiac vegetation along valvular coaptation lines without destruction of leaflets. NBTE vegetation is smaller and mostly detected by TEE, as in our case. Pathogenesis of NBTE is similar to the mechanisms underlying cancer hypercoagulability [7].

Patients with adenocarcinomas are reported to be at fivefold higher risk for nonbacterial endocarditis than patients with other histological types of malignant tumors [9]. NBTE, tumor embolism, neoplastic infiltration of cerebral vessels, and hypercoagulable conditions including hyperfibrinogenemia, hyperviscosity, and disseminated intravascular coagulation are the etiologies of stroke in cancer patients [10] and the nonbacterial endocarditis in the course of gynecological neoplasms (ovarian, cervix) is reported to have the highest potential to complicate with ischemic stroke [8].

Cerebral infarction from NBTE presents acutely with focal cerebral symptoms and signs that can be a transient ischemic attack or completed infarction. Multifocal ischemic events can occur. Aphasia is the most common neurologic sign and correlates with involvement of branches of the middle cerebral artery. In many patients, multifocal thromboembolism culminates in widespread infarctions of varying sizes, producing confusion and lethargy [11].

The treatment of NBTE includes anticoagulation and appropriate treatment of underlying cancer, although there is no established strategy for anticoagulation. A study performed using heparin versus warfarin in patients with NBTE and malignancy only showed benefit with heparin [12]. A large-scale prospective study confirmed that low-molecular-weight heparin is more effective than warfarin in preventing recurrent deep venous thrombosis and pulmonary embolism in cancer patients [13].

The hypercoagulability disorders in cancer patients could be resistant to routine anticoagulant treatment and clinicians, especially oncologists, may need to react faster than usual when dealing with primary oncological cases that have thrombotic conditions due to underlying neoplasms. When the hypercoagulable state in patients with ovarian cancer could not be totally cured by standard anticoagulation therapy with heparin or coumarin derivatives, trying to treat the underlying malignancy could help control the hypercoagulable status [14].

In conclusion, surgical resection of the primary tumor may prevent recurrent thrombotic events due to ovarian malignancy and may increase the effect of anticoagulant treatment. Oncologists should react promptly to break the circle of cancer, thrombosis, and inoperability.
